# Werner *et al.* Identification of Insulin-Like Growth Factor-I Receptor (IGF-IR) Gene Promoter-Binding Proteins in Estrogen Receptor (ER)-Positive and ER-Depleted Breast Cancer Cells

**DOI:** 10.3390/cancers2031642

**Published:** 2010-08-27

**Authors:** Rive Sarfstein, Antonino Belfiore, Haim Werner

**Affiliations:** 1 Department of Human Molecular Genetics and Biochemistry, Sackler School of Medicine, Tel Aviv University, Tel Aviv 69978, Israel; E-Mail: rives@post.tau.ac.il; 2 Department of Clinical and Experimental Medicine, University Magna Graecia of Catanzaro, Catanzaro 88100, Italy; E-Mail: belfiore@unicz.it

We have found a mistake in our paper recently published in *Cancers* [1]. Only one panel is shown for Figure 5 (page 251). A correct figure is provided here.

**Figure 5:**
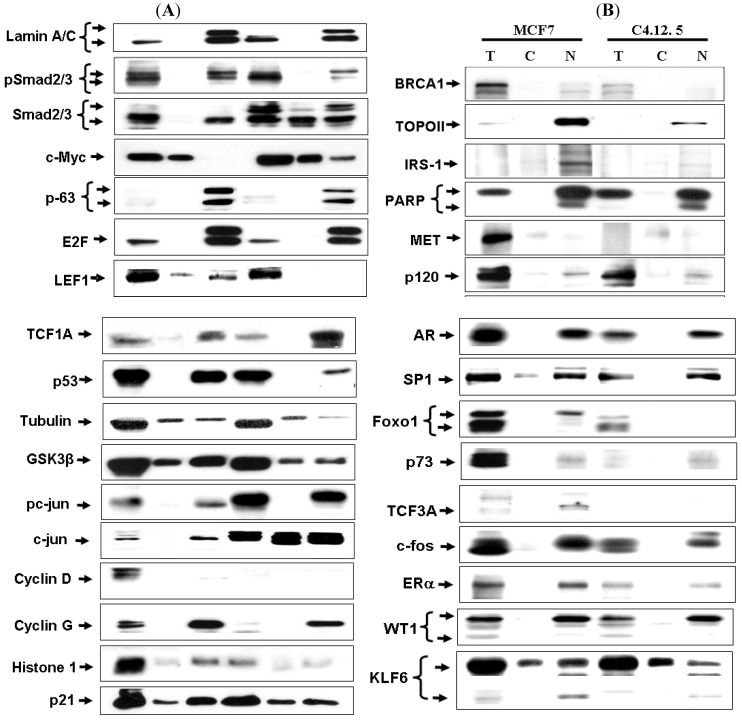
Cellular distribution of transcription factors in the MCF7 and C4.12.5 cell lines. Cell lines were fractionated as described under *Materials and Methods* and total lysates (T; 80 μg), cytosolic fractions (C; 20 μg), and nuclear extracts (N; 20 μg) were resolved on 10% SDS-PAGE and blotted with the indicated antibodies.
